# Stress Condition on a Restricted Sodium Diet Using Umami Substance (L-Glutamate) in a Pilot Randomized Cross-Over Study

**DOI:** 10.3390/foods10081739

**Published:** 2021-07-28

**Authors:** Tamami Iwamoto, Andrea Wakita, Saiko Shikanai, Hideki Matsumoto, Mariko Hirota, Hisayuki Uneyama, Vu Thi Thu Hien, Shigeru Yamamoto

**Affiliations:** 1 Department of Food and Nutritional, Faculty of Human Life, Jumonji University, Saitama 352-8510, Japan; yamamotoshigeru426@gmail.com; 2 Ajinomoto Co., Inc., Kanagawa 210-8681, Japan; wakita_andrea@ajinomoto.com (A.W.); hideki_matsumoto@ajinomoto.com (H.M.); mariko_hirota@ajinomoto.com (M.H.); hisayuki_uneyama@ajinomoto.com (H.U.); 3 Asian Nutrition and Food Culture Research Center, Saitama 352-8510, Japan; saiko_shikanai@yahoo.co.jp; 4 National Institute of Nutrition, Hanoi 84, Vietnam; hienvuthithu.nin@gmail.com

**Keywords:** stress, umami substances, restricted sodium diet, cross-over

## Abstract

Hypertensive patients who adopt a sodium-restricted diet have difficulty maintaining this change, and this could increase stress. On the other hand, soup rich in umami substances (dashi) was reported to reduce indexes of anxiety and stress. The objective of this study was to measure mood and physiological stress indexes during administration of a sodium-restricted diet with and without an umami substance (free L-glutamate) by a cross-over randomized, single-blind, placebo-controlled trial in Japanese female university students. The baseline was measured for 5 days followed by a sodium-restricted diet intervention phase that lasted for 10 days. The Profile of Mood States questionnaire was administered, a stress marker in saliva (chromogranin-A) was measured, and the amount of sodium intake was confirmed from 24 h urine collection samples. Results showed that the sodium reduction was verified by 24 h urine excretion. The percentage of change in the stress marker from the baseline showed that the stress level in group without the umami substance was significantly higher than that in the group with the umami substance (*p* = 0.013) after receiving a sodium-reduced diet for 6 or more days, indicating that stress was alleviated. This study suggested that umami substances might help to ameliorate stress during a sodium-reduced diet, especially in the initial phase.

## 1. Introduction

COVID-19, a new type of infectious disease, reminded us that prevention of noncommunicable diseases (NCDs) is an issue that we should solve beyond the boundaries of emerging and developing countries [1]. In 2013, WHO established the NCD Global Monitoring Framework and set seven goals for NCD prevention [2]. Among them, the only specific nutrient intake target is salt reduction, and it calls on member countries to aim for a 30% reduction in salt intake from 2011 to 2025. Since then, many initiatives on salt reduction have been launched, accelerating the global salt-reduction movement. However, as of 2020, no country has achieved this goal [3]. For instance, the Japanese National Nutrition and Health Survey 2019 reported that even though salt intake in Japanese was reduced from 10.7 to 10.1 g/day during the period 2009–2019 [4], the intake is still almost twice as much as the WHO recommendations of less than 5 g per day [5].

The latest analysis report of the Global Burden of Disease (GBD) has just been published in The Lancet. As of 2019, primary care management and reductions in salt intake are known to be potentially effective in reducing the burden of critical risk factors [6]. However, salt not only improves the taste of food but is also used to preserve and flavor foods and to improve yeast fermentation in dough [7,8,9], making a reduction a major challenge for the food industry. The committee on strategies to reduce sodium intake in the United State suggested the possibility of replacing some of the salt in food with other tastes or flavors [10]. An example is the addition of umami taste ingredients, dominantly composed of a building block of dietary protein, L-glutamate (an amino acid). Free L-glutamate can maintain food palatability with a lowered overall sodium amount in a food item [11,12,13,14]. In fact, free L-glutamate is being used in sodium-restricted diets such as for schizophreniform patients in hospital [15]. On the other hand, Kawano et al. have demonstrated that hypertensive patients who receive a sodium-restricted diet after diagnosis have difficulty maintaining the diet [16]; thus, in these subjects, having to continue with the diet could increase stress. 

Previous studies [17,18,19,20] showed improvement in mood state when subjects have an intake of dried bonito broth (dashi), which is rich in umami substances. For instance, in a randomized placebo-controlled human trial, it was found that dashi improved mood states and improved concentration significantly [17]. Another study reported that dashi not only improved the mood state but also decreased 8-hydroxy-2′-deoxyguanosine, an oxidative stress marker [19]. This evidence may indicate the possibility of umami taste substances having a positive benefit for healthy eating. Several authors have reported the relationship between diet and stress; most of these studies are on the effects of psychological/physiological stress on dietary choices [21,22] but not on the effect of having several days of a sodium-reduced diet on stress in human studies. Our hypothesis is that an umami substance could be used as an approach to enhance the taste and palatability of a sodium-restricted diet and reduce psychological and physiological stress. Therefore, we investigated the effects of sodium-reduced-diet intake on the measurement of mood using the Profile of Mood States (POMS) questionnaire and measured physiological stress by testing the concentration of a saliva sympathetic system marker, chromogranin-A (CgA pmol/mg protein), during a sodium-restricted diet with and without the addition of umami substances in the foods. It is well known that salivary CgA (pmol/mg protein) changes more rapidly and more sensitively to psychological stressors than salivary cortisol does [23,24], and it has the advantage for the participants of being a noninvasive method.

## 2. Materials and Methods

This study was approved by the Ethics Committees of Jumonji University (2012-008 and 2012-008-2), and Ajinomoto Co., Inc. (2012-008). Written consent was obtained from all subjects. This study was registered at UMIN-CTR Clinical Trial (UMIN000020514).

### 2.1. Subjects

Thirty-one Japanese female university students were recruited by voluntary participation. Women between 18 and 35 years old, with a regular menstrual cycle, who were not pregnant or lactating, without a habit of exhausting exercise, without chronic diseases, and without food allergies participated in the study.

### 2.2. Study Design

It was a cross-over randomized, single-blind, placebo-controlled trial. Participants were randomly divided into group A or group B and assigned to start a sodium-restricted diet without an umami substance as the control group (group A) or a sodium-restricted diet with an umami substance as the Glu group (group B) for ten days. Before dividing into the two groups, participants received a diet with more than 4000 mg of sodium for 5 days as the baseline phase to standardize the sodium intake before the intervention phase. This amount of sodium was close to the Japanese diary intake [4].

### 2.3. Diet Intervention

During the 5-day baseline and 10-day intervention phases, researchers provided participants with breakfast, lunch, dinner, snacks, and beverages. For snacks and beverages, the same brand of each item was purchased. Participants took the whole meals to eat at home or university, and they were instructed to eat all their food during the baseline and intervention phases, respectively. Snacks were optional. They were given a diary file to record all uneaten food portions (example: 1/4 main dish not eaten), snacks eaten, approximate amount of urine missed during collection, and comments regarding the study or whether their mood/physical condition was different from usual. The meals for lunch and dinner for the baseline and intervention were on 4-day menu cycles. The meals for breakfast and snacks were prepared or bought by Jumonji University. Main and side dishes for lunch and dinner were prepared for each participant in individual bags by the food factory Anshin Koubo Ltd., Yamagata, Japan, following standardized recipes and stored at −20 °C. The main and side dish portions were the same for all participants, but a dietitian adjusted individual dietary energy requirements by increasing or decreasing the amount of plain white rice and snacks. Energy and nutrients were calculated using the Excel Eiyokun software Ver. 6.0 (Kenpakusha Co., Ltd., Tokyo, Japan), based on the table of nutrient contents published by the Japanese Science and Technology Agency (5th edition). Before starting the study, randomized food samples from all diets (baseline, sodium-restricted diet without and with umami substance) were taken to analyze free L-glutamate, sodium, and potassium content at the Ajinomoto Co., Inc., laboratory. The free L-glutamate was measured by an amino acid analyzer (Hitachi L-8800 amino acid analyzer, Tokyo, Japan). Free L-glutamate intake by participants was calculated according to the data obtained at the laboratory of Ajinomoto Co., Inc. Sodium and potassium in the food were analyzed by inductively coupled plasma–atomic emission spectroscopy (ICPS-8100, Shimadzu). 

During the baseline, subjects received meals with sodium content higher than 4000 mg per day. In the intervention phase, subjects received a diet close to 2000 mg of sodium per day with a low amount of free L-glutamate intake for the control group, and high free L-glutamate for the umami group. The amounts of sodium and free L-glutamate used in the foods were controlled by the quantity of substances used, such as salt and umami seasonings, respectively. Subjects were not allowed to drink alcohol, coffee, or soft drinks with caffeine during the study. 

Before starting with the study, participants practiced all the procedures for taking samples (such as urine and saliva) and filling out the questionnaire (POMS).

### 2.4. Profile of Mood States (POMS) 

The full version of the POMS questionnaire consists of 65 items that are used to evaluate the mood state as follows: tension–anxiety, depression, anger–hostility, vigor, fatigue, and confusion. Subjects assessed moods they experienced the previous week on a five-point scale (0: not at all; 1: a little; 2: moderately; 3: quite a bit; 4: extremely frequent). The POMS—Japanese version published by Kaneko Shobo Co, Ltd. (Tokyo, Japan) was used as the test sheet. 

For the baseline, mood was assessed on the morning of day 1 (D1) and the morning of D1 of the intervention. These data were considered as the last data for the baseline phase. Since POMS reflects the mood state of the last week, for the intervention phase, it was conducted on the morning of D6 and the following morning after the completion of the intervention. 

### 2.5. Anthropometric Data

Height was self-reported using as reference the annual health check conducted at Jumonji University. Body weight, body muscle, and fat were measured by a bioelectrical impedance analysis system (InBody 2.0, Biospace Co., Ltd., Seoul, Korea). Subjects were asked to wear light clothing and to try to wear the same or similar clothes at each measurement. In addition, they removed shoes and socks to be measured by the InBody machine, they raised their arms 45° from the body and lightly pressed the metallic button until the measurement was completed. During the baseline phase, measurements were taken at D1 and the morning of D1 of the intervention. These data were considered as the last data for the baseline phase. For the intervention phase, anthropometric measurements were carried out on day 6 (D6) and the following morning after the completion of the intervention.

### 2.6. Saliva Sampling and Chromogranin-A Analyses

Saliva samples were collected between 7 and 9 a.m. before breakfast. For baseline, it was collected on D1 and the morning of D1 of the intervention as the last data for the baseline phase. During the intervention phase, the samples were taken on day 3 (D3), D6, day 8 (D8), and the following morning after the completion of the intervention.

Saliva samples were obtained using a saliva collector; the subjects chewed the cotton for three minutes, and then deposited it in a sample tube (Salivette, Sarstedt, Germany). The tubes were stored at −20 °C until centrifugation (3000 ppm, 5 min at 5 °C), and after centrifugation, they were stored again at −80 °C. The concentration of salivary chromogranin (Cg)-A (pmol/mg protein) was determined by Yanaihara Institute Inc. (Shizuoka, Japan). 

### 2.7. Twenty-Four-Hour Urine Collection

Urine samples were collected by subjects with special devices, the U-Container, a precise urine measurement device by Akita Sumitomo Bakelite Co., Ltd. (Akita, Japan), which is easy to handle. The first urine of the day was discarded, and collection was started from the second collection until the 24 h finish time. In case of a missing urine sample, participants had to record in their diary file the approximate amount of urine missed during the collection. The 24 h urine samples were taken on D1 and D5 of the baseline and D5 and D10 for the intervention phase. After finishing collection of the sample for the day, subjects brought it to Jumonji University, where it was stored at 5 °C, the total amount per day was calculated, and two samples of 10 mL were taken and stored at −20 °C until analysis. The amounts of sodium and potassium in the urine were analyzed at a laboratory testing service (Biomedical Laboratories, BML, Inc. (Saitama branch, Tokyo, Japan).

### 2.8. Statistical Analysis

Data are presented as means and standard deviations (SD), and they were analyzed at baseline and, for the sodium-restricted diets, for D1–5 and D6–10 in the control group and the Glu group. Levene’s test was used to test homogeneity of variance. A paired-sample t-test was conducted to compare means of energy and nutrient intakes during the baseline and the sodium-restricted diet of the control group, and the baseline and sodium-restricted of the Glu group; and to compare anthropometric measurements between the control and the Glu groups for D1–5 and D6–10. The stress marker (CgA pmol/mg protein) was calculated as the percentage of change from baseline to the intervention phase D1–5 or D6–10 for both sodium-restricted groups. Differences between D1–5 and D6–10 within groups, and differences between the control and the Glu groups for D1–5 and D6–10 were conducted using a paired sample t-test.

For urine and potassium excretion and POMS, a repeated-measure MANOVA was conducted to compare changes from baseline to D1–5 and for D6–10 followed by Tukey HSD post hoc test by controlling the random effect of the subjects when MANOVA showed differences. In data for which Levene’s test showed heterogeneity of variance, Wilcoxon *t*-test and Kruskal–Wallis (urine sodium excretions) were used. All statistical analyses were performed using JMP^®^ 14.2.0 (SAS Institute Inc., Cary, NC, USA). A value of *p* < 0.05 was considered significant. For statistical analysis, “n” was the sum sample size.

## 3. Results

**Figure 1:**
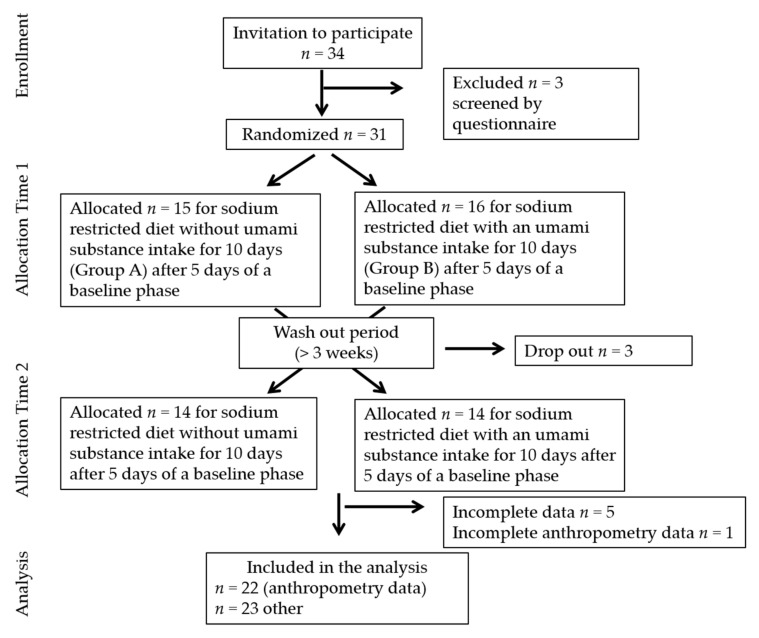
CONSORT diagram showing the flow of participants through each stage of the study.

Of the total 31 students, the stress marker and POMS analyses were performed on 23 participants (Figure 1), because 3 dropped out after developing flu during the intervention phase and 5 subjects had incomplete data. Anthropometric data, of only 22 subjects were considered because of incomplete data. The adherence to the diet was assessed by urinary sodium excretion, daily record forms, and face-to-face interviews each time participants picked up meals to take home or brought the urine samples to Jumonji University.

The dietary composition of sodium, potassium, and free L-glutamate is shown in Table 1, according to the food analyses at the laboratory of Ajinomoto Co., Inc. The baseline diet had 4612 ±177 mg/d of sodium, and the sodium-restricted diet of the control group had 1993 ± 235 mg/d and the Glu group had 2048 ± 250 mg/d of sodium.

Table 2 shows the energy and nutrient intake during the baseline and during both sodium-restricted diets calculated by Excel Eiyokun software. Free L-glutamate intake was calculated according to the data obtained at the laboratory of Ajinomoto Co., Inc. (Kanagawa, Japan)

The energy intake was lower in the sodium-restricted diet of the control group (7310 ± 905 kJ/day, *p* = 0.006) and the Glu group (7332 ± 923 kJ/day, *p* < 0.026) when these were compared with the baseline diet (7502 ± 819 kJ/day). Energy intake decreased with a slight reduction of protein (the control group) and fat intake (the control and the Glu groups).

The amount of urine excreted decreased from baseline to D1–5 and during D6–10 for both sodium-restricted diets (Table 3). The sodium was reduced significantly from baseline to D1–5 (52%) and to D6–10 (44%). The potassium excreted in urine decreased slightly in the control group (Table 3).

Anthropology data showed a slight difference between the sodium-restricted diet in the control and the Glu groups in BMI on D1–5 (*p* = 0.027) and in muscle amount on D6–10 (*p* = 0.047) (Table 4). Other anthropometric variables did not show a difference between the two sodium-restricted diet groups.

**Figure 2:**
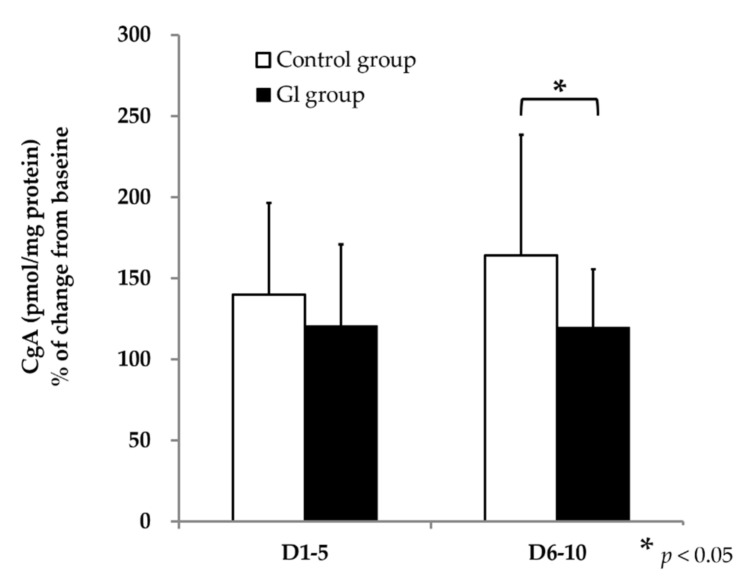
Stress condition marker (CgA pmol/mg protein) represented as percentage (%) of change from baseline to D1–5 or D6–10 in the sodium-restricted diet control group (white) and Glu group (black) and its standard deviation. In D6–10 CgA (pmol/mg protein) value as stress condition showed that Glu group value was significantly lower than control group diet by paired sample *t*-test (*n* = 23) (*p* = 0.013).

The stress marker (CgA pmol/mg protein) calculated as the percentage of change from baseline to the intervention phase D1–5 or D6–10 was significantly higher in the sodium-restricted diet of the control group in phase D6–10 than in the sodium-restricted diet of the Glu group (*p* = 0.013) (Figure 2). The difference between D1–5 and D6–10 was that the stress marker tended to increase in the control group, but it was not significant statically (*p* = 0.052).

POMS did not show any difference during either sodium-restricted diet (Supplementary Table S1).

## 4. Discussion

This (pilot) study suggests an important finding for healthy eating. In the late stage of the sodium-reduced diet (D6–D10), the change in saliva concentration of chromogranin-A was significantly higher than the baseline in the control group (Figure 2). On the other hand, the amount of sodium excretion in urine dropped during the intervention phase with the sodium-restricted diet (Table 3). This sodium reduction in the body by the continuous intake of a sodium-restricted diet could induce psychological stress. However, this psychological stress appears only as a physiological response, in the CgA (pmol/mg protein) and not in the POMS questionnaire, so its conscious effect may be weak. To our knowledge, this is the first study to show that having a sodium-reduced diet for several days can induce a physiological stress response as shown by our control group. Kawano et al. indicates that hypertensive patients have a higher salt craving when receiving a sodium-reduced diet than healthy individuals without sodium restrictions [16], so this may be worth confirming through a similar study in hypertensive patients. In our study, when an umami substance was added in the food, psychological stress was lower than in the control group with a sodium-reduced diet. Since the latest brain science studies using NIRS have shown that umami substances enhance salty-like acceptance [25], many scientists have shown that umami substances enhance the taste of sodium-reduced foods in different food groups [11,26,27,28,29]. Based on this evidence, the increase in psychological stress due to sodium-reduced-diet intake is probably related to the fact that it is difficult to switch to a sodium-reduced diet from a normal diet (due to factors such as a decrease in palatability). It seems that the acceptability of a low-salt diet can be increased with umami fortification to reduce psychological stress.

Decreased palatability due to a decrease in the “deliciousness” of a low-salt diet not only makes it difficult for healthy people to adopt a diet with lower sodium intake to prevent health risks such as cardiovascular diseases, but it is also an issue in improving the quality of life (QOL) of hospitalized patients, especially in malnourished elderly patients. In a recent study, sodium reduction in hospital meals showed a lower eating rate for hospitalized elderly patients, leading to poor nutritional status [30]. It has been shown that even in hospitalized patients, the eating rate of inpatients’ meals (sodium-reduced diet) in the psychiatric elderly is improved by the addition of umami seasonings to daily meals [15]. Positive usage of umami substances in a sodium-reduced diet is likely to be more helpful in improving the nutrition of inpatients.

In our study, the energy intake was decreased by a slight reduction of protein (control group) and fat intake (control and Glu groups) during intervention, but these reductions may not have been enough to produce a physiological effect, since the reductions were 192 and 162 kJ/d in the sodium-restricted control and Glu groups compared to baseline, respectively. We suggest that in the sodium-restricted diet the need to balance the tastes of salty foods with plain white rice could be reduced, and as consequence, the subjects could not finish the whole serving of rice and some side dishes. Jeffery et al. found that participants preferred to decrease the intake of sodium by increasing naturally lowsodium foods, such as fruits and vegetables, than by eating low-sodium processed foods, which were considered less palatable [31]. In our study, the processed foods were eaten for breakfast. These foods kept their original sodium content. The sodium intake from the processed food, and therefore its sodium, was controlled by instructing subjects about the amount of food to consume before starting the study. The amounts of free glutamate in the sodium-restricted control and Glu groups were controlled by the addition of sources of free glutamate, such as umami seasoning.

The 24 h urinary sodium excretion showed a reduction from the baseline to the intervention phase, which could suggest some physiological changes in the body in adapting to this reduction of sodium, such as increases in plasma renin activity and aldosterone [32]. The adherence to the diet was assessed by urinary sodium, daily record forms, and face-to-face interviews each time participants picked up meals to take home or brought the urine samples to Jumonji University. We could demonstrate that, in our population, a sodium-restricted diet caused stress as measured by the stress markers, but mood did not change as measured by POMS, though it has been reported by Ishizaki and Kuroda that dashi, which is rich in umami substances, could improve the mood state as measured by POMS [17,18,19].

As mentioned above, CgA (pmol/mg protein) increased in the sodium-restricted diet control group but did not change in the sodium-restricted diet Glu group. This could suggest that participants of the sodium-restricted diet Glu group could adapt better to the diet, as Mattes reported as a reason for accepting a lower-sodium diet [33], but these adaptations could occur physiologically without changing their mood. Our hypothesis concerning the increase in CgA (pmol/mg protein) in the sodium-restricted control group is that even though most of the participants did not realize which meals were the ones with umami seasoning, the information is not detected consciously but maybe it was perceived by the glutamate receptors in the mouth and in the gastro-intestinal tract [34,35] and transmitted to the brain; hence, it may not change the amount of the stress marker during the sodium-restricted diet of the Glu group. It has been suggested that after taste cognition in the cortex of the brain, taste information is further processed in the limbic system in terms of emotion. The outcome is finally conveyed to the hypothalamus for the regulation of feeding [36]. In an animal study, it was reported that the oral administration of 2% of dashi for 29 days reduced anxiety, which could be the result of changing amino acid levels in the brain [37]. In our study, the variability of CgA (pmol/mg protein) (Figure 2) was slightly large; however, the paired-sample *t*-test showed a strong significant difference between the control and the Glu group (*p* = 0.013). A previous study in young healthy subjects showed a large standard deviation [38]. CgA may vary among subjects; however, it has two advantages: it is a noninvasive test, and each participant can take the saliva sample at home.

To our knowledge, this is the first study carried out in humans to study the effect of umami seasoning on stress during a reduced-sodium diet. However, the study has some limitations. First, there was no questionnaire item regarding diet acceptability or sensory difference of the sodium-restricted control and Glu groups. However, during interviews, participants commented that they did not perceive the differences between the two sodium-reduced diets. Second, it is possible that students could have had an academic examination during the intervention phase, which could influence stress and mood state. However, this was a cross-over study, thus this bias should be minimized. Third, subjects were Japanese female students. Food preferences can be significantly influenced by food culture, and it may be necessary to consider whether the results of female students raised in the Japanese food culture rich in umami sources are applicable to Western food culture. However, this limitation may not be significant because the finding that the sodium-reduced foods’ palatability is improved by fortifying with umami taste is reported by several studies in different subjects from different food cultures [39,40,41,42].

## 5. Conclusions

The present study suggested that an umami substance, free L-glutamate, could ameliorate stress during a reduced-sodium diet. To achieve the maximum amount of sodium suggested by WHO without changing the stress indexes, free L-glutamate could be a tool to encourage consumers to make healthier choices without sacrificing palatability. These results may contribute to the development of strategies to achieve the sodium-reduction goals by using seasonings rich in umami taste.

## Figures and Tables

**Table 1:** Characteristics of the diet for the baseline and the sodium-restricted diets.

		Baseline Diet		Sodium-Restricted Diets			
				Control Group		Glu Group	
		Mean	SD	Mean	SD	Mean	SD
Free L-Glutamate	g/day	0.61	0.02	0.36	0.06	1.10	0.65
Sodium	mg/day	4612	177	1993	235	2048	250
Potassium	mg/day	2807	656	2149	209	1984	37

Values are mean and the standard deviation (SD) of the free-L-glutamate, sodium, and potassium in the food. Breakfast and snacks are included. Control group: sodium-restricted diet without umami substance. Glu group: sodium-restricted diet with umami substance (L-glutamate).

**Table 2:** Energy and nutrient intakes during baseline and the sodium-restricted diets.

		Baseline Diet		Sodium-Restricted Diet			
				Control Group		Glu Group	
		Mean	SD	Mean	SD	Mean	SD
Energy	kJ/day	7502	819	7310	905 **	7334	923 *
Protein	g/day	77	4	76	3 *	76	4
Fat	g/day	47	2	45	1 **	45	1 **
Carbohydrate	g/day	267	44	262	50	263	50
Free L-Glutamate	g/day	0.9	0.1	0.3	0.0 **	1.5	0.1 **

Values are mean and standard deviation (SD) (*n* = 23). Statistical analyses were performed by paired-sample *t*-test between baseline and the control group, and baseline and the Glu group. * *p* < 0.05, ** *p* < 0.01.

**Table 3:** Amount of urine, sodium, potassium, and chloride excreted in the baseline and in the sodium-reduced diets.

		Baseline		Sodium-Restricted Diet (Control Group)				Baseline		Sodium-Restricted Diet (Glu Group)			
				D1–5		D6–10				D1–5		D6–10	
		Mean	SD	Mean	SD	Mean	SD	Mean	SD	Mean	SD	Mean	SD
Urine	mL	1102	484	976	437	838	370 **	1084	441	997	415	881	434 *
Sodium	mg/day	2282	879	1149	464 **	981	426 **	2176	704	1178	470 **	958	383 **
Potassium	mg/day	1369	435	1345	509	1168	441 *	1348	420	1272	441	1363	544
Chlorine	mg/day	4013	1533	2073	868 **	1817	772 **	3839	1310	1948	892 **	1639	641 **

Values are mean and standard deviations (SD) (*n* = 23). Statistical analyses were performed by repeated-measures MANOVA followed by Tukey HSD when statistically significant differences were found. * *p* < 0.05, ** *p* < 0.01.

**Table 4:** Anthropometric measurements during the sodium-restricted diets.

		D1–5				*p*-Value	D6–10				*p*-Value
		Control Group		Glu Group			Control Group		Glu Group		
		Mean	SD	Mean	SD		Mean	SD	Mean	SD	
BMI	kg/m^2^	20.7	2.3	20.5	2.2	0.027	20.6	2.2	20.4	2.2	0.053
Body water	kg	25.1	3.3	25.0	3.0	0.286	25.3	3.2	25.1	3.1	0.039
Muscle	kg	34.3	4.5	34.2	4.1	0.391	34.5	4.3	34.3	4.2	0.047
Body fat	kg	14.7	3.7	14.2	3.7	0.095	14.2	3.5	13.9	3.6	0.246
Body fat	%	28.5	4.9	27.8	4.9	0.163	27.9	4.5	27.4	4.8	0.304

Values are mean and standard deviations (SDs) (*n* = 22). Statistical analyses were performed by paired-sample *t*-test. Of 31 subjects, 3 dropped out, and 6 subjects’ data were deleted because of incomplete data.

## Data Availability

This study was registered at UMIN-CTR Clinical Trial (UMIN000020514).

## Supplementary Materials

The following are available online at https://www.mdpi.com/article/10.3390/foods10081739/s1, Table S1: POMS score during baseline and sodium restricted diet.
