# Supplementary material for: Stress Condition on a Restricted Sodium Diet Using Umami Substance (L-Glutamate) in a Pilot Randomized Cross-Over Study
Source: Foods. 2021 Jul 28;10(8):1739. doi: 10.3390/foods10081739 (PMC8393573; doi:10.3390/foods10081739)
Supplement: Supplementary file 1 [file foods-10-01739-s001.zip › foods-1287769 - suppl.pdf]

**Table S1. POMS score during baseline and sodium restricted diet**

| | Baseline | | Sodium restricted diet (control group) | | | | Baseline | | Sodium restricted diet (Glu group) | | | |
|---|---|---|---|---|---|---|---|---|---|---|---|---|
| | | | D1-5 | | D6-10 | | | | D1-5 | | D6-10 | |
| **POMS scores** | **Mean** | **SD** | **Mean** | **SD** | **Mean** | **SD** | **Mean** | **SD** | **Mean** | **SD** | **Mean** | **SD** |
| Tension-anxiety | 48.2 | 10.4 | 47.5 | 10.2 | 48.3 | 10.4 | 50.3 | 8.0 | 49.3 | 10.0 | 48.7 | 10.7 |
| Depresion-dejection | 50.3 | 10.0 | 49.0 | 8.9 | 50.5 | 10.6 | 50.9 | 7.6 | 50.2 | 9.2 | 49.6 | 9.5 |
| Anger-hostility | 45.6 | 7.8 | 44.8 | 7.6 | 46.1 | 9.4 | 45.7 | 6.0 | 46.0 | 7.6 | 45.5 | 8.1 |
| Vigor-activity | 49.9 | 10.1 | 48.6 | 10.2 | 49.3 | 10.9 | 47.7 | 8.3 | 46.0 | 9.0 | 49.1 | 10.5 |
| Fatigue-inertia | 47.4 | 9.5 | 47.4 | 8.4 | 48.6 | 11.0 | 48.6 | 8.7 | 49.4 | 9.8 | 47.1 | 10.2 |
| Confusion-bewilderment | 52.5 | 11.8 | 51.5 | 10.9 | 51.9 | 11.4 | 54.3 | 9.8 | 54.7 | 9.2 | 52.1 | 10.5 |

Values are mean and standard deviations (SD) ($n$ = 23). Non-significant different were found among baseline and intervention neither between the control and the Glu groups.
